# The influence of timing of coronary angiography on acute kidney injury in out-of-hospital cardiac arrest patients: a retrospective cohort study

**DOI:** 10.1186/s13613-022-00987-w

**Published:** 2022-02-11

**Authors:** Gladys N. Janssens, Joost Daemen, Jorrit S. Lemkes, Eva M. Spoormans, Dieuwertje Janssen, Corstiaan A. den Uil, Lucia S. D. Jewbali, Ton A. C. M. Heestermans, Victor A. W. M. Umans, Frank R. Halfwerk, Albertus Beishuizen, Joris Nas, Judith Bonnes, Peter M. van de Ven, Albert C. van Rossum, Paul W. G. Elbers, Niels van Royen

**Affiliations:** 1 Department of Cardiology, Amsterdam Cardiovascular Sciences, Amsterdam UMC, Vrije Universiteit Amsterdam, De Boelelaan 1117, 1081HV Amsterdam, The Netherlands; 2 Department of Cardiology, Erasmus MC, ‘s Gravendijkwal 230, 3015CE Rotterdam, The Netherlands; 3 Department of Intensive Care Medicine, Erasmus MC, Gravendijkwal 230, 3015CE Rotterdam, The Netherlands; 4 Intensive Care Medicine, Maasstad Hospital, Maasstadweg 21, 3079DZ Rotterdam, The Netherlands; 5 Department of Cardiology, Noordwest Ziekenhuisgroep, Wilhelminalaan 12, 1815JD Alkmaar, The Netherlands; 6 Thoraxcentrum Twente, Medical Spectrum Twente, Koningsplein 1, 7512KZ Enschede, The Netherlands; 7 Department of Intensive Care, Medical Spectrum Twente, Koningsplein 1, 7512KZ Enschede, The Netherlands; 8 Department of Cardiology, Radboud University Medical Center, Geert Grooteplein Zuid 10, 6525GA Nijmegen, The Netherlands; 9 Department of Epidemiology and Biostatistics, Amsterdam UMC, Vrije Universiteit Amsterdam, De Boelelaan 1089a, 1081HV Amsterdam, The Netherlands; 10 Department of Intensive Care Medicine, Amsterdam University Medical Centre, location VUmc, Amsterdam, The Netherlands

**Keywords:** Acute kidney injury, Out-of-hospital cardiac arrest, Coronary angiography, Creatinine, Risk factors, Reperfusion injury

## Abstract

**Background:**

Acute kidney injury (AKI) is a frequent complication in cardiac arrest survivors and associated with adverse outcome. It remains unclear whether the incidence of AKI increases after the post-cardiac arrest contrast administration for coronary angiography and whether this depends on timing of angiography. Aim of this study was to investigate whether early angiography is associated with increased development of AKI compared to deferred angiography in out-of-hospital cardiac arrest (OHCA) survivors.

**Methods:**

In this retrospective multicenter cohort study, we investigated whether early angiography (within 2 h) after OHCA was non-inferior to deferred angiography regarding the development of AKI. We used an absolute difference of 5% as the non-inferiority margin. Primary non-inferiority analysis was done by calculating the risk difference with its 90% confidence interval (CI) using a generalized linear model for a binary outcome. As a sensitivity analysis, we repeated the primary analysis using propensity score matching. A multivariable model was built to identify predictors of acute kidney injury.

**Results:**

A total of 2375 patients were included from 2009 until 2018, of which 1148 patients were treated with early coronary angiography and 1227 patients with delayed or no angiography. In the early angiography group 18.5% of patients developed AKI after OHCA and 24.1% in the deferred angiography group. Risk difference was − 3.7% with 90% CI ranging from − 6.7 to − 0.7%, indicating non-inferiority of early angiography. The sensitivity analysis using propensity score matching showed accordant results, but no longer non-inferiority of early angiography. The factors time to return of spontaneous circulation (odds ratio [OR] 1.12, 95% CI 1.06–1.19, *p* < 0.001), the (not) use of angiotensin-converting enzyme inhibitor or angiotensin II receptor blocker (OR 0.20, 95% CI 0.04–0.91, *p* = 0.04) and baseline creatinine (OR 1.05, 95% CI 1.03–1.07, *p* < 0.001) were found to be independently associated with the development of AKI.

**Conclusions:**

Although AKI occurred in approximately 20% of OHCA patients, we found that early angiography was not associated with a higher AKI incidence than a deferred angiography strategy. The present results implicate that it is safe to perform early coronary angiography with respect to the risk of developing AKI after OHCA.

**Supplementary Information:**

The online version contains supplementary material available at 10.1186/s13613-022-00987-w.

## Background

In patients who achieve return of spontaneous circulation (ROSC) after out-of-hospital cardiac arrest (OHCA), the subsequent high morbidity and mortality are mostly due to neurologic injury, systemic ischemia–reperfusion injury and multi-organ dysfunction. Although post-resuscitation treatment for OHCA patients has improved over the years, prognosis for these patients remains poor. Several key predictors for survival after the event have been identified, and include witnessed arrest, early initiation of cardiopulmonary resuscitation, time to ROSC and control of acute kidney injury (AKI) [1]. AKI is often observed after cardiac arrest and associated with the occurrence of chronic kidney disease and adverse outcome [2–6]. Whether cardiac arrest patients develop AKI depends on several factors such as age, comorbidity, prior renal insufficiency and time to ROSC [5, 7]. Other risk factors include diabetes and hypertension [8–10]. Congestive heart failure, hypotension after cardiac arrest, female sex and the usage of an intra-aortic balloon pump are also associated with increased incidence of AKI [9].

Another factor that might influence the development of AKI after cardiac arrest is the usage of iodinated contrast during imaging techniques such as coronary angiography [11]. Currently, a delayed as opposed to immediate angiography is recommended to be considered in OHCA patients without ST-elevation. In the decision process when to perform angiography, the absence of an increased AKI incidence would permit both strategies. At present, it is unclear whether the incidence of AKI increases after the administration of iodinated contrast agents post-cardiac arrest and whether the timing of administration influences the development of AKI. Therefore, the main goal of this study was to test the hypothesis that in patients who are successfully resuscitated after cardiac arrest, a strategy of early coronary angiography would not be worse than a strategy of deferred angiography with respect to AKI.

## Methods

### Study design

Using data from 19 of 23 Dutch cardiac intervention centers, a retrospective cohort study of patients resuscitated after OHCA between January 2009 and July 2018 was conducted. The study protocol was approved by the IRB of the VU University Medical Center and ethics approval was obtained by the medical ethics committee of the VU University Medical Center, Amsterdam, the Netherlands, with approval number 2017.398. Informed consent was waived, as it was not achievable to obtain this from all patients of the large retrospective study cohort.

### Patients

Patients admitted to the participating hospitals and coded with OHCA were screened for inclusion in the study. Patients were eligible for this study if they were 18 years or older and had ROSC. Patients were excluded when they had no creatinine values until day 3 or if time from OHCA to angiography was unknown.

### Treatment

Post-resuscitation care was at the discretion of the clinicians responsible for the patient. According to international guidelines, in patients with persistent ST-elevation on the electrocardiogram (ECG) emergency angiography and subsequent percutaneous coronary intervention (PCI) was performed [12, 13]. In patients without ST-elevation, angiography was either performed immediately or delayed [14, 15]. Patients receiving angiography within 2 h after OHCA were considered to have had early angiography.

### Data collection and outcomes

Patient data were anonymized by assigning a random record number to each patient included in the study. Information regarding demographics, pre-hospital settings, status at admission, medical history, clinical and laboratory data (e.g., renal function, blood cell count, pH, lactate) was collected from patient records. Also, available renal function tests at 1 month and 1 year were collected.

The primary endpoint of this study was the AKI incidence in patients receiving early angiography compared to in patients with delayed or no angiography. AKI was defined according to the well-established Acute Kidney Injury Network (AKIN) criteria, ranging from stage 1 to 3, with higher stages indicating more severe renal failure (see Additional file 1) [16–18]. We used the creatinine level at admission as baseline value. Daily serum creatinine was measured in local laboratories. The highest creatinine value within 48 h after hospital admission was used to calculate the AKIN category to evaluate the effect of OHCA and, if performed, early angiography on kidney function. Because urine output per day but not per hour was collected, we used these additional data to classify AKIN in an additional analysis.

### Statistical analysis

Minimal sample size was calculated for the expected incidence and difference of AKI between the early and deferred angiography group. The previous study by Petek et al. showed an overall incidence of AKI of 15% with a (non-significant) difference of 4.3% between early and non-early angiography [19]. With an expected AKI incidence of 20% in both groups and a non-inferiority margin of 5% for the risk difference, we would need 792 patients per treatment group to have 80% power assuming non-inferiority testing at a one-sided significance level of 5%.

Primary non-inferiority analysis was done by calculating the risk difference with its 90% confidence interval (CI) using a generalized linear model for a binary outcome and the identity link-function. As a sensitivity analysis, we repeated the primary analysis using propensity score matching. Patients who underwent early angiography were matched with patients with deferred or no angiography, according to their propensity score. The propensity score was defined as the probability that a patient would be assigned to a treatment (early or deferred angiography) based upon the clinically plausible explanatory variables including age, sex, comorbidity (i.e., diabetes mellitus, chronic kidney disease, hypertension), serum creatinine level, witnessed cardiac arrest, Glasgow Coma Scale < 8, first rhythm and signs of ST-elevation myocardial infarction (STEMI) on the ECG. Propensity scores for early vs. delayed or no angiography were estimated using a match tolerance of 0.1.

Continuous variables were compared using the independent-samples *t*-test for normally distributed data and summarized by mean and standard deviation. Skewed data were compared using the Mann–Whitney *U* test and summarized by median and interquartile range. Categorical variables were compared using the Chi-square test or Fisher’s exact test in case of low expected cell counts and summarized by counts and percentages.

Univariable and multivariable logistic regression analysis was used to identify predictors of AKI. Statistical significance was assumed when *p*-values for two-sided testing were < 0.05, except for the primary non-inferiority hypothesis for which a *p*-value for one-sided testing of < 0.05 was considered significant. Further details of the statistical analysis are provided in Additional file 1.

## Results

### Patients

**Fig. 1:**
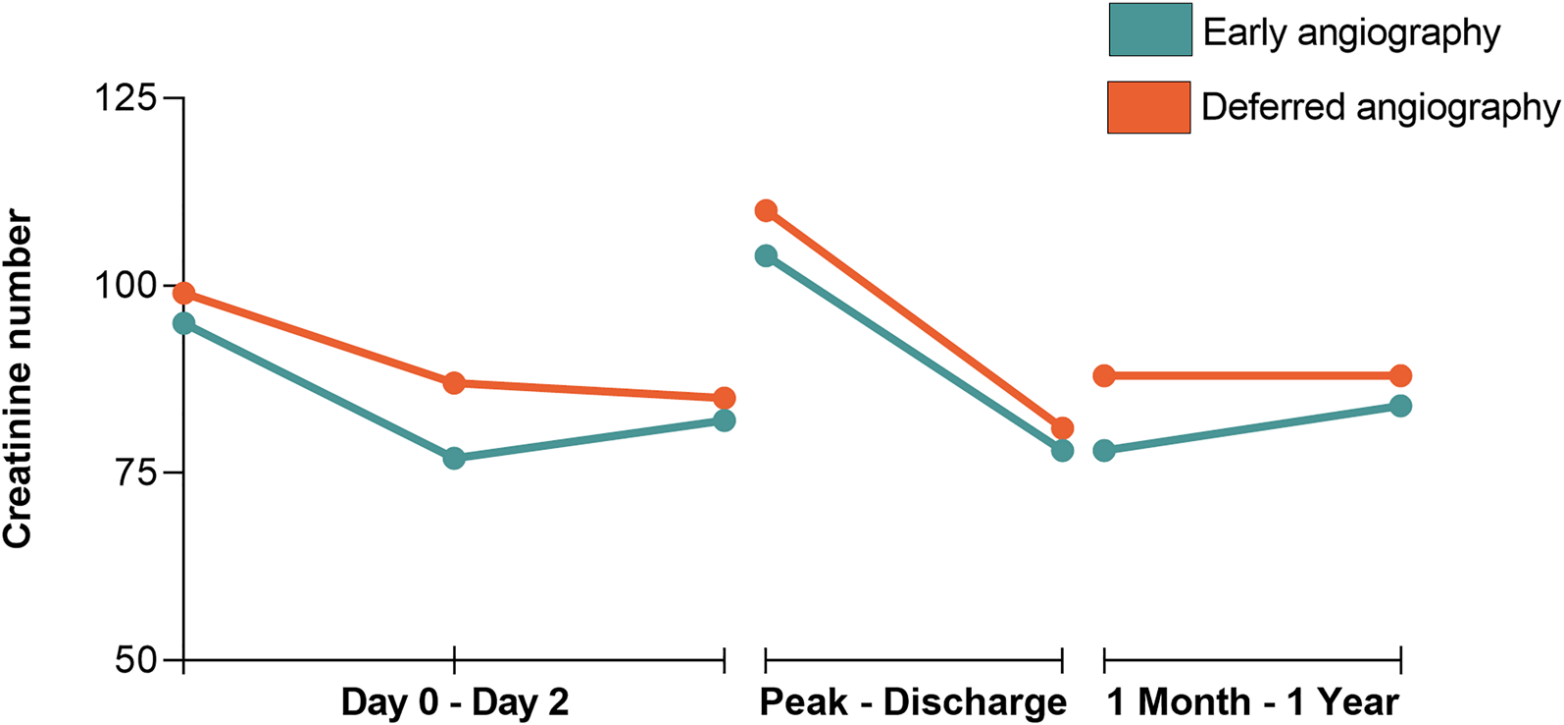
Creatinine levels. *CAG* coronary angiography

A total of 2375 OHCA patients were included in this study. Of these patients 1148 were treated with early angiography and 1227 with delayed or no angiography (Additional file 1: Fig. S1). Most patients were male (69.6%) (Table 1). Patients in the deferred angiography group were slightly older than patients in the early angiography group (62.7 ± 12.5 vs. 64.0 ± 14.9, *p* = 0.02). In both groups ventricular tachycardia/ventricular fibrillation was the most common arrest rhythm, but more frequently in the early angiography group (93.2% vs. 75.5%, *p* < 0.001). Overall, the median time to ROSC was 15 min (10–23). Patients in the deferred angiography group were more frequently comatose on admission (92.1% vs. 86.9%, *p* = 0.001), while patients in the early angiography group more often had a STEMI [12] at medical presentation (42.3% vs. 13.4%, *p* < 0.001). In the deferred angiography group, more patients had chronic kidney disease (8.8% vs. 3.4%, *p* < 0.001) and accordingly their median creatinine values at admission were higher than in the early angiography group (Table 1, Fig. 1, Additional file 1: Table S1). A comparison of baseline characteristics of the matched patients can be found in Additional file 1: Table S2.

**Table 1:** Baseline characteristics

Characteristics	All patients (*N* = 2375)	Early CAG (*N* = 1148)	Deferred/no CAG (*N* = 1227)	*P* value
Patient characteristics				
Male sex	1652 (69.6)	791 (47.9)	861 (52.1)	0.50
Age in years	63.3 ± 13.8	62.7 ± 12.5	64.0 ± 14.9	0.02
Hypertension	839/2041 (41.1)	411/966 (42.5)	428/1075 (39.8)	0.21
Diabetes mellitus	379/2219 (17.1)	180/1109 (16.2)	199/1110 (17.9)	0.29
Hypercholesterolemia	288/1211 (23.8)	128/530 (24.2)	160/681 (23.5)	0.88
Previous cardiac arrest	21/1003 (2.1)	9/577 (1.6)	12/426 (2.8)	0.17
Previous myocardial infarction	495/2189 (22.6)	231/1100 (21.0)	264/1089 (24.2)	0.07
Previous CVA or TIA	43/690 (6.2)	24/357 (6.7)	19/333 (5.7)	0.64
Chronic kidney disease	69/1141 (6.0)	20/586 (3.4)	49/555 (8.8)	< 0.001
Previous renal replacement therapy	15/1009 (1.5)	7/579 (1.2)	8/430 (1.9)	0.40
Pre-hospital characteristics				
Arrest witnessed	981/1227 (80.0)	495/608 (81.4)	486/619 (78.5)	0.20
First rhythm				< 0.001
VF/VT	1009/1193 (84.6)	572/614 (93.2)	437/579 (75.5)	
PEA	57/1193 (4.8)	12/614 (2.0)	45/579 (7.8)	
Asystole	127/1193 (10.6)	30/614 (4.9)	97/579 (16.8)	
Time from arrest to BLS in minutes	3 [1–5]	3 [1–5]	3 [1–5]	0.40
Time from arrest to ROSC in minutes	15 [10–23]	15 [10–23]	15 [10–23]	0.69
Characteristics on hospital arrival				
Glasgow Coma Scale < 8	1374/1533 (89.6)	639/735 (86.9)	735/798 (92.1)	0.001
Signs of STEMI on ECG	543/1965 (27.6)	410/969 (42.3)	133/996 (13.4)	< 0.001
CT scan performed	320/751 (57.4)	205/378 (54.2)	115/373 (30.8)	< 0.001
Laboratory values upon admission				
pH	7.2 [7.1–7.3]	7.2 [7.1–7.3]	7.2 [7.1–7.3]	0.05
Lactate, mmol/L	5.9 [3.5–9.1]	5.5 [3.5–8.8]	6.2 [3.7–9.4]	0.01
Hemoglobin, mmol/L	8.4 ± 1.3	8.5 ± 1.2	8.3 ± 1.3	0.001
Hematocrit	0.40 ± 0.06	0.41 ± 0.1	0.39 ± 0.1	0.001
Leukocytes, ·10^9^/L	12.5 [9.8–16.7]	12.6 [10.0–17.0]	12.2 [9.4–16.1]	0.07
CRP, mg/L	5 [3–17]	4 [3–16]	5 [3–19]	0.15
Creatinine, µmol/L	98 [82–117]	96 [81–114]	100 [82–120]	0.001
Creatinine > 130 µmol/L	366/2342 (15.6)	148/1134 (12.9)	218/1208 (17.8)	0.001

All data are expressed in proportions of the population with known data and percentages (%). Plus–minus (±) values are classified as mean and standard deviation (SD). Brackets are classified as median and interquartile ranges (IQR)

*CAG* coronary angiography, *CVA* cerebrovascular accident, *TIA* transient ischemic attack, *VF* ventricular fibrillation, *VT* ventricular tachycardia, *PEA* pulseless electrical activity, *BLS* basic life support, *ROSC* return of spontaneous circulation, *STEMI* ST-segment elevation myocardial infarction, *CT* computed tomography, *CRP* C-reactive protein

### Treatments

Angiography was performed in 48.9% of patients with deferred angiography (Table 2). Median time to angiography was 75 (52–93) minutes in the early angiography group and 1003 (151–8795) minutes in the delayed angiography group (*p* < 0.001), with a maximum time frame depending on length of intensive care unit stay. PCI was performed in 82.7% of the patients in the early angiography group and in 36.2% of the deferred angiography group (*p* < 0.001). Furthermore, patients in the early angiography group underwent a computerized tomography (CT) scan more frequently than patients in the deferred angiography group (54.2% vs. 30.8%, *p* < 0.001). Approximately 80% of patients in both groups were treated with targeted temperature management.

**Table 2:** In-hospital procedures, treatments and characteristics

	Early CAG group (*N* = 1148)	Deferred CAG/no CAG group (*N* = 1227)	*p*-value
Targeted temperature management	670/849 (78.9)	570/709 (80.4)	0.47
Hypotension	244/554 (44.0)	293/692 (42.3)	0.55
Use of inotropic or vasopressors	319/512 (62.3)	422/688 (61.3)	0.73
Use of intra-aortic balloon pump	96/993 (9.7)	40/1026 (3.9)	< 0.001
Heart failure < 45%	200/496 (40.3)	134/313 (42.8)	0.48
Need for renal replacement therapy	34/839 (4.1)	42/701 (6.0)	0.08
Major bleeding^a^	15/347 (4.3)	23/357 (6.4)	0.21
CAG performed	1148 (100.0)	600/1227 (48.9)	< 0.001
PCI performed	205/248 (82.7)	129/356 (36.2)	< 0.001
CABG	8/292 (2.7)	9/392 (2.3)	0.71
Laboratory values			
Lowest pH	7.2 [7.1–7.3]	7.2 [7.0–7.3]	0.003
Peak lactate, mmol/L	5.5 [3.4–9.1]	6.0 [3.4–9.5]	0.38
Peak CRP, mg/L	105 [30–201]	122 [61–200]	0.06
Peak leukocytes, ·10^9^/L	18.4 [14.3–24.2]	17.6 [13.9–23.2]	0.34
Peak CK, U/L	1604 [629–4421]	828 [296–2411]	< 0.001
Survival at hospital discharge	544/765 (71.1)	596/850 (70.1)	0.66

All data are expressed in proportions of the population with known data and percentages (%). Brackets are classified as median and interquartile ranges (IQR)

*CAG* coronary angiography, *PCI* percutaneous coronary intervention, *CABG* coronary artery bypass graft, *CRP* C-reactive protein, *CK* creatine kinase

^a^Major bleeding was scored using BARC classification scores ≥ 3, ranging from bleeding that is not actionable to fatal bleedings

### Acute kidney injury

**Fig. 2:**
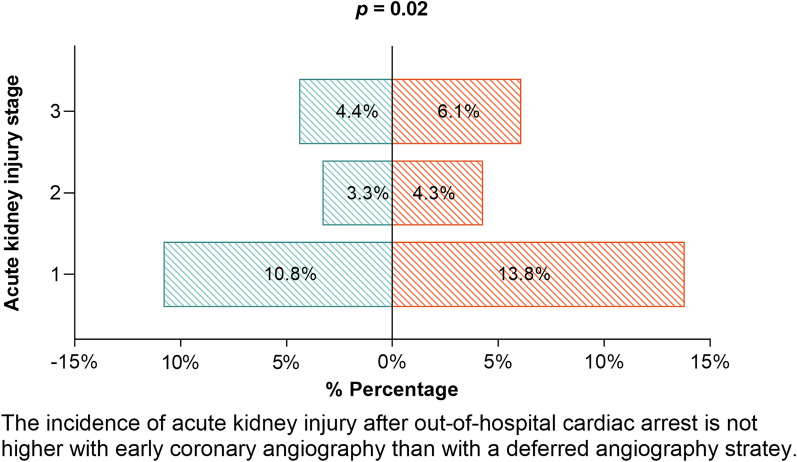
Incidence of acute kidney injury. On the left side in the graph the early angiography group is displayed in blue, on the right side the non-early angiography group is displayed in red

In the early angiography group 18.5% of patients and in the deferred angiography group 24.1% developed AKI after OHCA (*p* = 0.002). Risk difference was − 3.7% with 90% CI − 6.7 to − 0.7%, indicating non-inferiority of early angiography in terms of AKI after OHCA. When using urine output for defining AKI as well, this revealed higher proportions of AKI in both groups with the same trend favoring early angiography (early angiography 30.2% vs. deferred angiography 36.5%, *p* = 0.002) (Additional file 1: Table S3). After propensity score matching, the proportion that developed AKI after OHCA was 24.6% in the early angiography group and 27.5% in the deferred angiography group (*p* = 0.61). Risk difference was − 3.3% with 90% CI ranging from − 12.8 to 6.3%, favoring early angiography, but no longer indicating non-inferiority of early angiography at the prespecified margin of 5% (Table 3). The composite endpoint of AKI and mortality within 48 h showed similar results (early angiography 25.5% vs. 32.2% in delayed angiography, *p* = 0.001). Most frequently patients developed AKIN stage 1 (10.8% in early angiography vs. 13.8% in the deferred angiography group, *p* = 0.02) (Fig. 2, Additional file 1: Table S4). Overall, 3.8% of patients developed AKI stage 2 and 5.3% AKI stage 3. Propensity matched analysis showed that the distribution in AKI stages did not differ (Table 3). In both groups, in patients who developed AKI, this occurred most frequently already at day 1 (early angiography group 69.4% and deferred angiography group 73.6%). Within the deferred angiography group, patients without angiography developed more AKI than patients undergoing late angiography (24.4% vs. 18.8%, *p* = 0.037). Renal replacement therapy (RRT) was not performed more frequently in the early angiography group (4.1% vs. 6.0%, *p* = 0.08) (Table 2). Because RRT could have been used for other reasons than kidney failure, we evaluated AKI (stage 3) without the application of RRT as a criterion. Twenty-one patients had no AKI anymore after reclassifying. It did not significantly alter the outcomes (Additional file 1: Table S5). Patients undergoing a CT scan (with the use of contrast) did not develop AKI more frequently (Additional file 1: Table S6). Creatinine at day 3 was persistently higher in the deferred angiography group compared to in patients with early angiography (84 [67–126] µmol/L vs. 79 [65–102] µmol/L, *p* < 0.001) (Fig. 1, Additional file 1: Table S1). Creatinine before discharge and available creatinine values 1 month and 1 year after discharge were not different between the groups.

**Table 3:** Sensitivity analysis of acute kidney injury outcomes in matched patient cohorts

AKI outcome	Early CAG (*n* = 125)	Deferred/no CAG (*n* = 125)	*p*-value
AKI present	29/118 (24.6)	33/120 (27.5)	0.61
AKI stage			0.51
No AKI	89/118 (75.4)	87/120 (72.5)	
1	18/118 (15.3)	16/120 (13.3)	
2	3/118 (2.5)	8/120 (6.7)	
3	8/118 (6.8)	9/120 (7.5)	
Survival at hospital discharge	84/125 (67.2)	84/125 (65.2)	1.00

All data are expressed in proportions of the population with known data and percentages (%)

*AKI* acute kidney injury, *CAG* coronary angiography

Patients who developed AKI were characterized by older age, a greater proportion of diabetes, a lower incidence of witnessed arrest and shockable rhythm, a longer duration from arrest to basic life support and ROSC, more frequently hypotension, heart failure and treatment with inotropics or vasopressors and targeted temperature management (Additional file 1: Table S7).

Logistic regression analysis was performed to identify predictors of AKI (Table 4). Time to ROSC (odds ratio [OR] 1.12, 95% CI 1.06–1.19, *p* < 0.001), the (not) use of an angiotensin-converting enzyme inhibitor (ACEI) or angiotensin II receptor blocker (ARB) (OR 0.20, 95% CI 0.04–0.91, *p* = 0.04) and baseline creatinine (OR 1.05, 95% CI 1.03–1.07, *p* < 0.001) were identified as independent predictors for AKI. After correction for risk factors, early coronary angiography and amount of contrast were not found to be associated with the occurrence of AKI.

**Table 4:** Univariable and multivariable analysis on predictors of acute kidney injury

Predictors and covariates	Univariable analysis		Multivariable analysis*	
	Odds ratio (95% CI)	*p*-value	Odds ratio (95% CI)	*p*-value
Early coronary angiography	0.71 (0.58–0.88)	0.002		
Sex (reference = female)	1.07 (0.85–1.35)	0.57		
Age (per year)	1.01 (1.00–1.02)	0.03		
Diabetes mellitus	1.36 (1.03–1.78)	0.03		
Hypertension	0.96 (0.77–1.21)	0.74		
Arrest witnessed	1.35 (0.95–1.94)	0.10		
Time from arrest to BLS (per minute)	1.07 (1.03–1.11)	< 0.001		
Time from arrest to ROSC (per minute)	1.03 (1.02–1.04)	< 0.001	1.12 (1.06–1.19)	< 0.001
Targeted temperature management	1.92 (1.28–2.88)	0.002		
Use of ACE-inhibitor or ARB	1.97 (1.43–2.70)	< 0.001	0.20 (0.04–0.91)	0.04
Heart failure (LVEF < 45%)	1.57 (1.09–2.26)	0.02		
Hypotension > 30 min	3.19 (2.43–4.18)	< 0.001		
Contrast used (per 10 mL)	1.05 (1.03–1.08)	< 0.001		
Creatinine at baseline (per 1 pnt)	1.01 (1.01–1.01)	< 0.001	1.05 (1.03–1.07)	< 0.001
Use of IABP	1.06 (0.69–1.64)	0.80		
First rhythm (reference = VF/VT)				
PEA	2.38 (1.29–4.42)	0.01		
Asystole	2.82 (1.87–4.25)	< 0.001		
Glasgow Coma Scale < 8	3.27 (1.63–6.54)	0.001		

This figure shows the independent predictors of acute kidney injury in patients successfully resuscitated of out-of-hospital cardiac arrest. Time to return of spontaneous circulation and creatinine at baseline were independent predictors for the presence of acute kidney injury

*ACE-inhibitor* angiotensin-converting enzyme, *ARB* angiotensin II receptor blocker, *BLS* basic life support, *IABP* intra-aortic balloon pump, *LVEF* left ventricular ejection fraction, *PEA* pulseless electrical activity, *ROSC* return of spontaneous circulation, *VF* ventricular fibrillation, *VT* ventricular tachycardia

*If *p*-value of univariable analysis was < 0.1, variables were entered in a backward multivariable analysis

### Mortality

Mortality within 48 h was 7.5% in the early angiography group and 11.2% in the deferred group. Survival until discharge was relatively high at 70.6% and did not differ between the groups, neither after propensity score matching (Tables 2 and 3). In patients with AKI stage 3, survival at discharge and 1 year was lower at 44.4%, respectively, 31.0%, both *p* < 0.001. Of the patients treated with RRT 47.0% survived until discharge (*p* = 0.006) and 38.6% were still alive after 1 year.

## Discussion

### Key findings

In OHCA patients, early angiography did not show a higher incidence or grade of AKI. These findings suggest that coronary angiography can be performed safely either early or delayed in patients after OHCA with regard to the development of AKI. This outcome is an important addition to the results of the randomized COACT trial, which showed a similar survival with immediate and delayed angiography in OHCA patients without STEMI and no difference in AKI between the groups [20]. Also, the present data are in line with findings from smaller retrospective cohort studies [19–22].

The reported incidence of AKI after cardiac arrest is up to 80% and depends on population and definition criteria [23]. The AKI incidence of 21.2% in our population is in line with previous results [19]. Baseline risk factors for AKI, such as an older age, might have contributed to the decision for deferral. Patients in the early angiography group more frequently had a shockable rhythm and subsequently ST-elevation on the ECG, while patients with deferred angiography were more frequently comatose and had higher levels of lactate on admission. This may reflect differences in cardiovascular performance between the groups, although the use of inotropics or vasopressors did not differ. Other baseline patient characteristics were balanced between the groups. Furthermore, in a patient-matched analysis no higher incidence of AKI was found in the early angiography group.

In accordance with previous, smaller studies, the timing of coronary angiography and amount of contrast were not associated with the development of AKI [19, 21, 22, 24]. The lack of association between early angiography and AKI occurrence in OHCA patients might partly be explained by the control of the ischemia–reperfusion phenomena as part of the post-cardiac arrest syndrome, that is clearly more beneficial than the potential risk of contrast in these patients [25]. Management of the post-cardiac arrest syndrome involves intensive care support, but also coronary angiography when there are signs of persistent ischemia (ST-elevation myocardial infarction) on the ECG to preserve cardiac contractility for an effective circulation. A higher baseline creatinine level, the use of an ACEI or ARB and prolonged time to ROSC were independent predictors for AKI. The latter may reflect the intensity of ischemia–reperfusion injury after OHCA, of which the association with AKI has been noted in several studies [5, 24, 26, 27]. The found protective effect of ACEI or ARB use for AKI highlights the importance of identification of patients at risk in order to reduce AKI development. Also increased creatinine on admission has been associated with development of AKI [5, 23, 24, 27].

We did not find a higher incidence of AKI with early angiography, but even a higher incidence of AKI in the deferred angiography group. Remarkably, patients without angiography developed more AKI than patients that did undergo angiography. This can be explained by the fact that in patients with an overall worse condition, angiography is more often delayed. This group was older and had more comorbidities. However, mortality in the delayed angiography group was not higher than in the early angiography group.

We observed an association between the development of severe AKI after OHCA and increased mortality, which is in agreement with previous reports [2, 5, 24, 26–29]. Patients who developed AKI more frequently needed treatment with an inotropic or vasopressor. The optimal balance between level of perfusion and vasoactive support with regard to renal function after cardiac arrest is unknown. On the one hand, increasing mean arterial pressure improves renal function in OHCA patients, but on the other hand a higher level of vasoactive support may increase the incidence of AKI [30]. This aspect of clinical management after cardiac arrest must be studied in future research. Targeted temperature management was used as much in the early as in the deferred angiography group, however, patients who developed AKI were more frequently treated with targeted temperature management than patients without AKI. In a substudy of the TTM trial no difference in the incidence of AKI was found between temperature management at 33 °C or 36 °C degrees [24]. Patients who received targeted temperature management in our study may have been in a worse clinical condition. Intended targeted temperature management may be a factor in the timing of coronary angiography, as in the COACT trial patients undergoing immediate angiography reached target temperature later than the delayed angiography group [20]. Although the optimal strategy for targeted temperature management is still unclear [31, 32], potential benefit from early cooling may be a reason to postpone angiography in comatose OHCA patients without STEMI [33].

One might question whether modern iodinated contrast media are nephrotoxic at all. Several studies concluded that early intervention is not harmful with regard to AKI [20–22, 26, 34]. A meta-analysis of three matched cohort studies suggested that no association between AKI and iodinated contrast exposure in critically ill patients exists at all [19, 35]. Iodinated contrast is thought to cause acute tubular necrosis through hypoxia from renal vasoconstriction and by direct cytotoxic effects within the nephron [36–38]. Although nephrotoxicity from iodinated contrast has been well established, little is known about the pathophysiology of AKI after cardiac arrest.

The absence of augmented renal injury after early coronary angiography can be a contribution to the management of patients after OHCA without STEMI, especially in the light of the newest international guidelines [39]. At present, delayed as opposed to immediate angiography is recommended to be considered in OHCA patients without ST-elevation. In the decision process when to perform angiography, the absence of an increased AKI incidence permits both strategies, individualized according to clinical status as well as to logistical circumstances.

### Limitations

To the best of our knowledge, this study is the first multicenter retrospective study to evaluate the association between timing of coronary angiography and AKI in a sizeable patient cohort. Nevertheless, several limitations of our study should be noted. First, causality of the results is difficult to determine and therefore the results should be interpreted with caution. Second, an immortal time bias exists, because patients who were included in the early or late angiography groups could not have died until their treatment. Third, data on pre-admission renal function were not available. The use of the first serum creatinine values on arrival might have underestimated prior renal function, resulting in a lower AKI incidence. However, we note that it may take 24–36 h for serum creatinine to rise after a definite renal insult [40]. Therefore, it is likely that the creatinine concentrations measured rapidly after OHCA are reasonably representative of pre-existing renal function. Furthermore, due to the retrospective study design, for several parameters, including survival and creatinine values at day 1 or 2, some data were missing, so AKI could have been missed. AKI was defined according to AKIN criteria because we did not have enough creatinine values from day 4–7 to apply the more recent KDIGO criteria. Due to the time window of 48 h after admission for creatinine measurements, we have no information on the incidence of AKI post-angiography in the deferred group. However, this does not influence the conclusion that early-angiography is not associated with an increased incidence of AKI in this cohort. Fourth, potentially, there might have been other nephrotoxic agents than iodinated contrast that could have induced AKI after OHCA. Finally, we cannot exclude that performance of early angiography was influenced by factors that also could impact the occurrence of AKI.

## Conclusions

Even though the incidence of AKI in OHCA patients was substantial in this large cohort study, we found that early angiography was not associated with a higher AKI incidence than a deferred angiography strategy. AKI incidence was even higher in the deferred angiography group, both before and after propensity score matching. We did not find an effect of coronary angiography on renal function when adjusted for other risk factors for AKI after OHCA. The present results implicate that it is safe to perform early coronary angiography with respect to the risk of developing AKI after OHCA.

## Supplementary Information

**Additional file 1.** AKI definition, Additional statistical analysis, Table S1-7, Figure S1.

## Data Availability

The datasets used during the current study are available from the corresponding author on reasonable request.

## Abbreviations

ACEI: Angiotensin converting enzyme inhibitor
AKI: Acute kidney injury
AKIN: Acute Kidney Injury Network
ARB: Angiotensin II receptor blocker
CAG: Coronary angiography
CI: Confidence interval
CRP: C-reactive protein
CT: Computerized tomography
ECG: Electrocardiogram
IQR: Interquartile range
OHCA: Out-of-hospital cardiac arrest
OR: Odds ratio
PCI: Percutaneous coronary intervention
ROSC: Return of spontaneous circulation
RRT: Renal replacement therapy
STEMI: ST-segment elevation myocardial infarction
VT/VF: Ventricular tachycardia/ventricular fibrillation
