# Supplementary material for: The influence of timing of coronary angiography on acute kidney injury in out-of-hospital cardiac arrest patients: a retrospective cohort study
Source: Ann Intensive Care. 2022 Feb 11;12:12. doi: 10.1186/s13613-022-00987-w (PMC8837770; doi:10.1186/s13613-022-00987-w)
Supplement: Supplementary file 1 — Additional file 1. AKI definition, Additional statistical analysis, Table S1-7, Figure S1. [file 13613_2022_987_MOESM1_ESM.docx]

Additional file 1

This appendix has been provided by the authors to give readers additional information about their work.

Supplement to: Janssens GN, Daemen J, Lemkes JS, et al. The influence of timing of coronary angiography on acute kidney injury in out-of-hospital cardiac arrest patients: a retrospective cohort study. Annals of Intensive Care.

**Acute kidney injury according to Acute Kidney Injury Network criteria**

Creatinine/glomerular filtration rate criteria

Stage 1: Increase in serum creatinine of more than or equal to 0.3 mg/dL (≥26.4 μmol/L) or increase to more than or equal to 150% to 200% (1.5- to 2-fold) from baseline.

Stage 2: Increase in serum creatinine to more than 200% to 300% (>2- to 3-fold) from baseline.

Stage 3*: Increase in serum creatinine to more than 300% (>3-fold) from baseline (or serum creatinine of more than or equal to 4.0 mg (≥354 μmol/L) with an acute increase of at least 0.5 mg/dl (44 μmol/L).

Urine output criteria

Stage 1: Urine output <0.5 ml/kg/h x 6 h

Stage 2: Urine output <0.5 ml/kg/h x 12 h

Stage 3: Urine output <0.3 ml/kg/h x 24 h or anuria x 12 h

* Patients who received renal replacement therapy (RRT) were considered to have met the criteria for stage 3 irrespective of the stage they were in at the time of RRT.

**Additional statistical analysis**

Univariable and logistic regression analysis was used to identify predictors of AKI. A multivariable model was subsequently determined using backward multivariable logistic regression analysis. Predictors with p-value <0.1 in univariable analysis, were included as candidate predictors in multivariate analysis.

Statistical analyses were performed using SPSS Statistics, version 26 (IBM Corp, Armonk, New York).

**Table S1. Creatinine values**

Medians and means of creatinine values throughout hospital admission, stratified by early and non-early CAG

| **Creatinine value** | **Early CAG group**  **(N=1148)** | **Deferred CAG/no CAG group**  **(N=1227)** | **p-value** |
| --- | --- | --- | --- |
| Creatinine at t=0, µmol/L | 96 [81-114] | 100 [82-120] | 0.001 |
| Creatinine at day 1, µmol/L | 78 [62-108] | 90 [68-130] | <0.001 |
| Creatinine at day 2, µmol/L | 82 [67-108] | 86 [67-137] | 0.008 |
| Creatinine at day 3, µmol/L | 79 [65-102] | 84 [67-126] | <0.001 |
| Last creatinine before hospital discharge | 79 [66-100] | 83 [66-117] | 0.08 |
| Peak creatinine during hospitalization, µmol/L | 105 [87-133] | 112 [89-170] | <0.001 |
| Creatinine 1 month after discharge (n=135), µmol/L | 78 [69-87] | 88 [68-102] | 0.05 |
| Creatinine 1 year after discharge (n=91), µmol/L | 84 [74-100] | 88 [74-105] | 0.68 |

Values are expressed as median and interquartile ranges (IQR).

CAG denotes coronary angiography, PCI percutaneous coronary intervention, CABG coronary artery bypass graft, CRP C-reactive protein, CK creatine kinase.

**Table S2. Characteristics in matched patients**

| **Characteristics** | **Early CAG**  **(N=125)** | **Deferred/ no CAG**  **(N=125)** | **P value** | **SD/agreement** |
| --- | --- | --- | --- | --- |
| Patient characteristics |  |  |  |  |
| Male sex | 98/125 (78.4) | 92/125 (73.6) | 0.37 | 95.2 |
| Age in years | 62.0±12.2 | 61.5±15.0 | 0.77 | 0.04 |
| Hypertension | 42/125 (33.6) | 39/125 (31.2) | 0.69 | 97.6 |
| Diabetes mellitus | 19/125 (15.2) | 19/125 (15.2) | 1.00 | 100.0 |
| Previous cardiac arrest | 3/125 (2.4) | 5/125 (4.0) | 0.72 | 98.4 |
| Previous myocardial infarction | 29/125 (23.2) | 26/125 (20.8) | 0.65 | 97.6 |
| Previous CVA or TIA | 18/247 (7.3) | 13/235 (5.5) | 0.46 | 97.3 |
| Chronic kidney disease | 3/125 (2.4) | 2/125 (1.6) | 1.00 | 99.2 |
| Previous renal replacement therapy | 0/125 (0.0) | 0/125 (0.0) | NA | 100.0 |
| Pre-hospital characteristics |  |  |  |  |
| Arrest witnessed | 102/125 (81.6) | 109/125 (87.2) | 0.22 | 94.4 |
| First rhythm |  |  | 0.29 | 96.0 |
| VF/VT | 100/125 (80.0%) | 105/125 (84.0) |  |  |
| PEA | 7/125 (5.6) | 2/125 (1.6) |  |  |
| Asystole | 18/125 (14.4) | 18/125 (14.4) |  |  |
| Time from arrest to BLS in minutes | 4 [2-7] | 3 [2-7] | 0.91 | 0.22 |
| Time from arrest to ROSC in minutes | 18 [15-25] | 20 [15-25] | 0.14 | 0.17 |
| Characteristics on hospital arrival |  |  |  |  |
| Glasgow coma scale <8 | 118/125 (94.4) | 123/125 (98.4) | 0.17 | 96.0 |
| Signs of STEMI on ECG | 43/125 (34.4) | 36/125 (28.8) | 0.34 | 94.4 |
| CT scan performed | 45/125 (36.0) | 78/125 (62.4) | <0.001 | 73.6 |
| Laboratory values upon admission |  |  |  |  |
| pH | 7.2 [7.1-7.3] | 7.2 [7.1-7.3] | 0.43 | 0.00 |
| Lactate, mmol/L | 6.0 [3.4-9.6] | 6.7 [4.4-9.9] | 0.22 | 0.17 |
| Hemoglobin, mmol/L | 8.6±0.89 | 8.7±1.2 | 0.36 | 0.12 |
| Hematocrit | 0.40±0.05 | 0.41±0.06 | 0.27 | 0.14 |
| Leukocytes, ∙10^9^/L | 13.2 [10.4-16.8] | 12.5 [10.2-16.5] | 0.61 | 0.13 |
| CRP, mg/L | 2.6 [2.5-6.3] | 2.5 [2.5-9.0] | 0.33 | 0.004 |
| Creatinine, µmol/L | 90 [74-110] | 98 [81-120] | 0.18 | 0.20 |
| Creatinine >130 µmol/L | 18/125 (14.4) | 19/125 (15.2) | 0.86 | 99.2 |

All data are expressed in proportions of the population with known data and percentages (%). Plus-minus (±) values are classified as mean and standard deviation. Brackets are classified as median and interquartile ranges (IQR). For continuous variables standardized difference is shown, for dichotomous variables agreement in percentage is shown.

CAG denotes coronary angiography, CVA cerebrovascular accident, ECG electrocardiogram, NA not applicable, TIA transient ischemic attack, VF ventricular fibrillation, VT ventricular tachycardia, PEA pulseless electrical activity, STEMI ST-segment elevation myocardial infarction, CT computed tomography, CRP C-reactive protein, SD standardized difference.

**Table S3. Acute kidney injury in patients according to creatinine levels and urine output**

| **AKI outcome** | **Early CAG (n=1128)** | **Delayed/no CAG (n=1178)** | **p-value** |
| --- | --- | --- | --- |
| AKI present | 310/1028 (30.2) | 402/1100 (36.5) | 0.002 |
| AKI stage |  |  | <0.001 |
| No AKI | 718/1028 (69.8) | 698/1100 (63.5) |  |
| 1 | 74/1028 (7.2) | 102/1100 (9.3) |  |
| 2 | 144/1028 (14.0) | 150/1100 (13.6) |  |
| 3 | 92/1028 (8.9) | 150/1100 (13.6) |  |

All data are expressed in proportions of the population with known data and percentages (%).

AKI denotes acute kidney injury, CAG coronary angiography.

**Table S4. Sensitivity analysis of acute kidney injury in patients without a history of RRT or chronic kidney disease (possible confounders for AKI)**

| **AKI outcome** | **Early CAG (n=1128)** | **Delayed/no CAG (n=1178)** | **p-value** |
| --- | --- | --- | --- |
| AKI present | 173/960 (18.0) | 246/1018 (24.2) | 0.001 |
| AKI stage |  |  | 0.01 |
| No AKI | 787/960 (82.0) | 772/1018 (75.8) |  |
| 1 | 100/960 (10.4) | 143/1018 (14.0) |  |
| 2 | 32/960 (3.3) | 44/1018 (4.3) |  |
| 3 | 41/960 (4.3) | 59/1018 (5.8) |  |

All data are expressed in proportions of the population with known data and percentages (%).

AKI denotes acute kidney injury, CAG coronary angiography.

**Table S5. Acute kidney injury in patients according to creatinine levels (application of RRT not taken into account)**

| **AKI outcome** | **Early CAG (n=1128)** | **Delayed/no CAG (n=1178)** | **p-value** |
| --- | --- | --- | --- |
| AKI present | 160/957 (16.7) | 236/1015 (23.3) | 0.001 |
| AKI stage |  |  | 0.001 |
| No AKI | 797/957 (83.3) | 779/1015 (76.7) |  |
| 1 | 108/957 (11.3) | 153/1015 (15.1) |  |
| 2 | 40/957 (4.2) | 51/1015 (5.0) |  |
| 3 | 12/957 (1.3) | 32/1015 (3.2) |  |

All data are expressed in proportions of the population with known data and percentages (%).

AKI denotes acute kidney injury, CAG coronary angiography.

**Table S6. Acute kidney injury in patients with or without coronary angiography and CT scan.**

| **AKI outcome** | **Early angiography + CT scan (N=161)** | **Early angiography, no CT scan (N=185)** | **Delayed angiography + CT scan (N=81)** | **Delayed angiography, no CT scan (N=50)** | **No angiography, CT scan (N=156)** | **No angiography or CT scan (N=48)** | **p-value** |
| --- | --- | --- | --- | --- | --- | --- | --- |
|  |  |  |  |  |  |  | 0.003 |
| AKI present | 34 (21.1) | 42 (22.7) | 13 (16.0) | 12 (24.0) | 58 (37.2) | 13 (27.1) |  |
| No AKI | 127 (78.9) | 143 (77.3) | 68 (84.0) | 38 (76.0) | 98 (62.8) | 35 (72.9) |  |

All data are expressed in numbers and percentages (%).

AKI denotes acute kidney injury, CT computed tomography,

**Table S7. Patient characteristics according to development of acute kidney injury**

| **Characteristics** | **AKI all stages**  **(N=374)** | **No AKI**  **(N=1523)** | **p-value** |
| --- | --- | --- | --- |
| Male sex | 265 (70.9) | 1059 (69.5) | 0.62 |
| Age in years | 64 ± 14 | 63 ± 14 | 0.038 |
| Hypertension | 136/337 (40.4) | 536/1303 (41.1) | 0.80 |
| Diabetes mellitus | 73/359 (20.3) | 222/1429 (15.5) | 0.029 |
| Hypercholesterolemia | 42/175 (24.0) | 176/755 (23.3) | 0.15 |
| Previous cardiac arrest | 7/185 (3.8) | 14/669 (2.1) | 0.19 |
| Previous myocardial infarction | 90/360 (25.0) | 296/1404 (21.1) | 0.11 |
| Previous CVA or TIA | 6/69 (8.7) | 25/417 (6.0) | 0.08 |
| Chronic kidney disease | 15/188 (8.0) | 36/752 (4.8) | 0.08 |
| Previous renal replacement therapy | 4/185 (2.2) | 7/674 (1.0) | 0.26 |
| Arrest witnessed | 146/197 (74.1) | 618/752 (82.2) | 0.012 |
| First rhythm |  |  | <0.001 |
| VF/VT | 141/194 (72.7) | 635/729 (87.1) |  |
| PEA | 14/194 (7.2) | 28/729 (3.8) |  |
| Asystole | 39/194 (20.1) | 66/729 (9.1) |  |
| Time from arrest to BLS in minutes | 5 [2-7] | 3 [1-5] | <0.001 |
| Time from arrest to ROSC in minutes | 20 [10-30] | 15 [10-20] | <0.001 |
| Targeted temperature management | 220/248 (88.7) | 783/976 (80.2) | 0.002 |
| Hypotension | 153/259 (59.1) | 269/804 (33.5) | <0.001 |
| Use of inotropic or vasopressors | 210/252 (83.3) | 446/777 (57.4) | <0.001 |
| Use of intra-aortic balloon pump | 23/352 (6.5) | 82/1266 (6.5) | 0.97 |
| Heart failure <45% | 67/137 (48.9) | 218/561 (38.9) | 0.032 |
| Need for RRT | 68/247 (27.5) | 0 (0.0) | <0.001 |
| Major bleeding* | 10/146 (6.8) | 18/457 (3.9) | 0.15 |

All data are expressed in proportions of the population with known data and percentages (%). Plus-minus (±) values are classified as mean and standard deviation (SD). Brackets are classified as median and interquartile ranges (IQR).

AKI denotes acute kidney injury, BLS basic life support, CVA cerebrovascular accident, PCI percutaneous coronary intervention, CABG coronary artery bypass graft, CRP C-reactive protein, CK creatine kinase, PEA pulseless electrical activity, ROSC return of spontaneous circulation, RRT renal replacement therapy, TIA transient ischemic attack VF ventricular fibrillation, VT ventricular tachycardia.

**
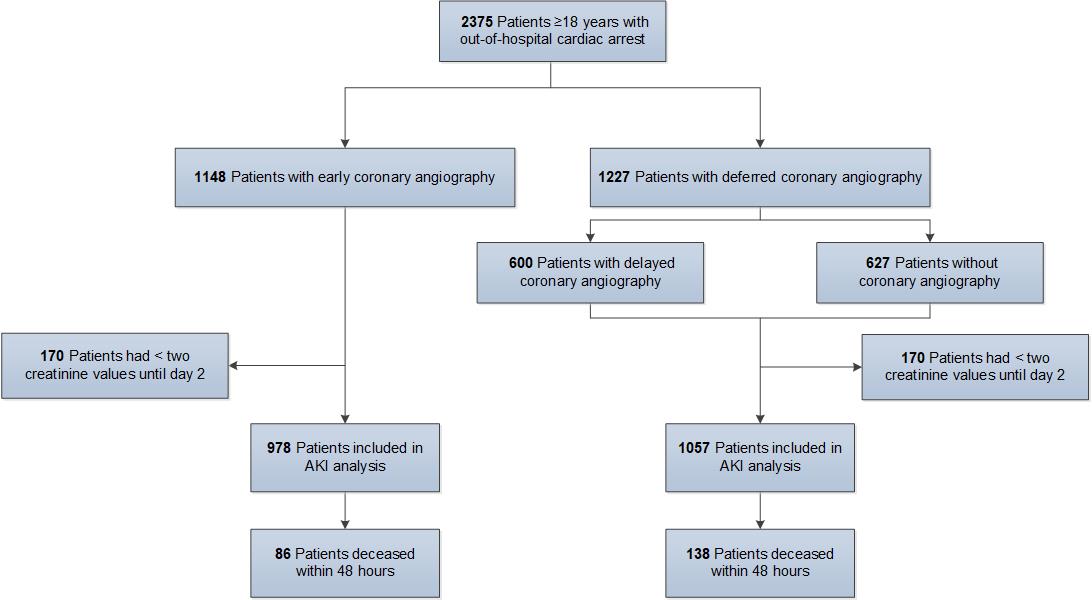
Figure S1. Study flowchart**
